# *NSD1* Mutations and Pediatric High-Grade Gliomas: A Comparative Genomic Study in Primary and Recurrent Tumors

**DOI:** 10.3390/diagnostics13010078

**Published:** 2022-12-27

**Authors:** Antonio d’Amati, Arianna Nicolussi, Evelina Miele, Angela Mastronuzzi, Sabrina Rossi, Francesca Gianno, Francesca Romana Buttarelli, Simone Minasi, Pietro Lodeserto, Marina Paola Gardiman, Elisabetta Viscardi, Anna Coppa, Vittoria Donofrio, Isabella Giovannoni, Felice Giangaspero, Manila Antonelli

**Affiliations:** 1 Anatomic Pathology Unit, Department of Emergency and Organ Transplantation, University of Bari, 70124 Bari, Italy; 2 Department of Molecular Medicine, University La Sapienza, 00161 Rome, Italy; 3 Department of Pediatric Onco-Hematology and Cell and Gene Therapy, Bambino Gesù Children’s Hospital, IRCCS, 00165 Rome, Italy; 4 Pathology Unit, Department of Laboratories, Bambino Gesù Children’s Hospital, IRCCS, 00165 Rome, Italy; 5 Department of Radiological, Oncological and Anatomo-Pathological Sciences, University La Sapienza, 00161 Rome, Italy; 6 Surgical Pathology Unit, Department of Medicine (DIMED), University Hospital of Padua, 35128 Padua, Italy; 7 Hematology Oncology Division, Department of Women’s and Children’s Health, University of Padova, 35128 Padua, Italy; 8 Department of Experimental Medicine, University La Sapienza, 00161 Rome, Italy; 9 Anatomic Pathology Unit, Santobono-Pausilipon Children’s Hospital, 80129 Naples, Italy; 10 IRCCS Neuromed, 86077 Pozzilli, Italy

**Keywords:** pediatric high-grade gliomas, hemispheric pediatric high-grade gliomas, high grade glioma H3-/IDH-wildtype, glioma, CNS tumors, molecular biology, WES, methylation profiling, *NSD1* gene, targeted therapy

## Abstract

Pediatric high-grade gliomas represent a heterogeneous group of tumors with a wide variety of molecular features. We performed whole exome sequencing and methylation profiling on matched primary and recurrent tumors from four pediatric patients with hemispheric high-grade gliomas. Genetic analysis showed the presence of some variants shared between primary and recurrent tumors, along with other variants exclusive of primary or recurrent tumors. *NSD1* variants, all novel and not previously reported, were present at high frequency in our series (100%) and were all shared between the samples, independently of primary or recurrence. For every variant, in silico prediction tools estimated a high probability of altering protein function. The novel *NSD1* variant (c.5924T > A; p.Leu1975His) was present in one in four cases at recurrence, and in two in four cases at primary. The novel *NSD1* variant (c.5993T > A; p.Met1998Lys) was present in one in four cases both at primary and recurrence, and in one in four cases only at primary. The presence of *NSD1* mutations only at recurrence may suggest that they can be sub-clonal, while the presence in both primary and recurrence implies that they can also represent early and stable events. Furthermore, their presence only in primary, but not in recurrent tumors, suggest that *NSD1* mutations may also be influenced by treatment.

## 1. Introduction

Pediatric high-grade gliomas (pHGGs) represent a heterogeneous group of neoplasms that preferentially arising in infant and children and accounting for one-third of pediatric gliomas, which includes circumscribed and diffuse gliomas with CNS-WHO (Central Nervous System-World Health Organization) grade 3–4. The understanding of biological alterations in these tumors has been transformed through the novel insights in the field of genome- and epigenome-wide molecular profiling techniques [1].

This approach allowed the redefinition of childhood gliomas and led to a reclassification from a morphology-based characterization to a molecular subgrouping. Collaborative molecular analyses have revealed that pHGGs have clear differences in location, age at presentation, clinical outcome, gender distribution, predominant histology and concurrent epigenetic and genetic alterations [2,3]. 

The distinct origins that underlie the different anatomical distribution of tumors, i.e., H3.3 G34R/V mutations, are found exclusively in the cerebral hemisphere. H3.3 K27M is found throughout the midline structures (including the thalamus, brainstem, cerebellum and spine) and H3.1 K27M is preferentially restricted to the pons [4,5,6]. In adolescents, IDH1 mutations represent a small proportion of cases [7].

To date, pHGGs also include diffuse pediatric-type high-grade gliomas H3-wildtype/IDH-wildtype, which have been recently subdivided into three molecular entities on the basis of DNA methylation profile. The subgroups are receptor tyrosine kinase type I (RTK I) and II (RTK II) and MYCN-amplified types [8,9]. These molecular subtypes have been associated with different outcomes: RTK II shows a significantly longer survival time, MYCN-amplified displays poor outcomes, while RTK I group harbors an intermediate prognosis [8,9]. Analysis of methylation patterns indicates that these three molecular subtypes are clearly distinct from the adult tumors [8].

Moreover, infant-type high-grade gliomas appear clinically distinct from their counterparts in older children and comprise novel subgroups with a prevalence of ALK, NTRK1-3, ROS1 and MET gene fusions. Kinase fusion-positive tumors show a better outcome and respond to targeted therapy, highlighting that histopathologic grading may not accurately reflect the biology of these tumors [10].

Finally, among the pHGGs group, there are also circumscribed tumors, such as anaplastic pleomorphic xanthoastrocytoma (CNS-WHO grade 3), which show characteristic molecular features represented by the BRAFV600E mutation and CDKN2A/B homozygous deletion [1,11].

Due to the genetic heterogeneity in pHGGs and considering the differences with the adult-type counterparts, it would be noteworthy to understand the chronological sequence of molecular changes, including the therapy-induced ones, in recurrent tumors. Additional genomic alterations could drive the growth of recurrences with a genomic profile that differs from the initial tumor and could be responsible for therapy failure in recurrent disease. Recent studies on spatially distinct tumor samples suggest that intratumoral heterogeneity should also be considered among factors responsible for treatment failure [12,13,14,15].

Recent large-scale sequencing studies have characterized the genomic landscape of untreated tumors. Johnson et al. [14] performed a whole exome sequencing (WES) of primary and recurrent adult-type diffuse gliomas and observed that some mutations present at diagnosis were not conserved at recurrence. Nevertheless, very few studies analyzed recurrent diffuse high-grade gliomas in pediatric age [16].

The aim of our study was to compare the genomic profile in a small series of hemispheric pediatric high-grade gliomas at initial diagnosis and recurrence. For this purpose, we selected four pairs of primary and recurrent supratentorial pediatric high-grade gliomas. In order to uncover genetic alterations and to dissect their genetic heterogeneity, we performed WES and DNA methylation profiling, comparing results of paired primary and recurrent tumors.

## 2. Materials and Methods

### 2.1. Clinical Cohort

The series included eight formalin-fixed paraffin-embedded (FFPE) tissue samples from four patients corresponding to four tumors’ pairs acquired from diagnosis as well as recurrence from 2008 and 2018.

Cases were retrieved as part of the Italian National Program of Centralization of Pediatric Brain Tumor (contributed by: Sapienza University of Rome (Rome, Italy), Fondazione Policlinico Universitario “A.Gemelli” IRCCS (Rome, Italy), Ospedale Pediatrico Bambino Gesù, (Rome, Italy), University of Padua (Padua, Italy).

The patient cohort was selected on the basis of the availability of material from both the primary and recurrent tumor for each case. To ensure adequate tumor content, hematoxylin and eosin (H&E) slides were reviewed after the initial cut of each FFPE block for DNA extraction. In three out of four patients (#1, #2, #3) residual tumor, both at primary and recurrence, was poorly represented for the presence also of normal cerebral tissue.

Histology was reviewed by two expert neuropathologists (FG and MA) in an independent manner. The study was performed according to local ethical and Institutional Review Board approval (Protocol number: 0102463).

### 2.2. Immunohistochemistry

Immunohistochemical (IHC) analysis was performed using a Leica Bond RXm™ automated staining processor (Leica Biosystems, Buffalo Grove, IL, USA). Tissue sections were cut at 5 μm, dried at 70 °C for 30 min and then dewaxed. Antigen retrieval was performed in the Bond Rx system with Epitope Retrieval Solution 1 (pH6) for 30 min. Sections were incubated for 30 min with Rabbit pAb to Histone H3 (di methyl K36) ChIP Grade (Abcam ab9049) (1:100).

Considering the small amount of residual tumor component in the two infant glioma cases (#1 and #3), we used pan-TRK IHC and ALK IHC as a screening tool for potential *NTRK* gene fusions and *ALK* gene alterations. Positive IHC expression with a cutoff of 1% should lead to an RNA-based NGS for detection/confirmation of the specific fusion in the appropriate clinical setting. We used the pan-TRK rabbit monoclonal antibody (clone EPR17341, RTU, Assay, Roche/Ventana) and ALK (clone D5F3/Ventana) on the Ventana BenchMark.

### 2.3. Whole Exome Sequencing (WES) Analysis

Next generation sequencing experiments, comprising DNA extraction and samples quality control, were performed by Genomix4life S.R.L. (Baronissi, Salerno, Italy). DNA was extracted from FFPE sections using AllPrep DNA/RNA FFPE Kit (Qiagen, Venlo, The Netherlands) according to manufacturer’s instructions. Indexed libraries were prepared from 100 ng/ea purified DNA with DNA Prep for Enrichment Kit with Illumina Exome Panel (45 M size) according to the manufacturer’s instructions. Libraries were quantified using the TapeStation 4200 (Agilent Technologies, Santa Clara, CA, USA) and Invitrogen Qubit fluorometer (Thermo Fisher Scientific, Waltham, MA, USA). They were then pooled such that each index-tagged sample was present in equimolar amounts, with a final concentration of the pooled samples of 2 nM. The pooled samples were subject to cluster generation and sequencing using an Illumina NextSeq 550 System (Illumina, San Diego, CA, USA) in a 2 × 150 paired-end format. Paired-end reads were aligned to the NCBI reference sequence (GRCh37/hg19) using the Borrows–Wheeler Algorithm (BWA) and variant calls were made using the Genomic Analysis Tool kit (GATK). With Picard tools in Java that work with next-generation sequencing data in BAM format, mean coverage information for every target can also be computed. The means coverage of at least 50× were obtained.

### 2.4. Data Analysis

All the variants with a MAF < 0.05 were visually examined and verified using integrative genomics viewer (IGV) version 5.01 (http://www.broadinstitute.org/igv (accessed on 3 September 2021)) and reported following the HGVS guidelines (http://www.hgvs.org/mutnomen/ (accessed on 12 September 2021)) on the basis of the coding sequences specifically indicated for each gene variant. Each variant was reported referring to the following databases: Clinical Variants (https://www.ncbi.nlm.nih.gov/pubmed (accessed on 5 September 2021)), dbSNP138, Leiden Open (source) Variation Database (LOVD) (http://www.lovd.nl/3.0/home (accessed on 5 September 2021)), COSMIC (https://cancer.sanger.ac.uk/cosmic (accessed on 5 September 2021)), Varsome (https://varsome.com/ (accessed on 7 September 2021)) and IARC TP53 database (https://p53.iarc.fr/ (accessed on 7 September 2021)).

To predict the possible impact of amino acid substitution on the protein structure and function, variants were evaluated by the following in silico tool predictors: SIFT-PROVEAN (http://provean.jcvi.org/index.php (accessed on 12 September 2021)), Polyphen-2 (http://genetics.bwh.harvard.edu/pph2/ (accessed on 12 September 2021)), GVGD (http://agvgd.hci.utah.edu/agvgd_input.php (accessed on 15 September 2021)) and MutationTaster (http://www.mutationtaster.org/ (accessed on 15 September 2021)). To predict possible impact on the splicing process by Fruit Fly Splice Predictor, NNSPLICE (http://www.fruitfly.org/seq_tools/splice.html (accessed on 18 September 2021)), Human Splicing Finder (http://www.umd.be/HSF3/ (accessed on 18 September 2021)), NetGene2 (http://www.cbs.dtu.dk/services/NetGene2/ (accessed on 20 September 2021)), ESEfinder3.0 (http://rulai.cshl.edu/cgi-bin/tools/ESE3/esefinder.cgi?process=home (accessed on 20 September 2021)), Alternative Splice Site Predictor ASSP (http://wangcomputing.com/assp/index.html (accessed on 23 September 2021)) and EX-SKIP (https://ex-skip.img.cas.cz/ (accessed on 27 September 2021)) were used. Only the variants with at least three deleterious predictions have been considered and reported. The structure and protein domain organizations of the proteins were obtained from the UniProt database (https://www.uniprot.org/ (accessed on 27 September 2021)).

### 2.5. DNA Methylation and Copy Number Variation Analysis

We performed DNA methylation profiling on samples of paired primary and recurrent pHGGs (with the limitation that the residual sample size was small). Neoplastic tissue from FFPE blocks was relevant (>70%) for patient #4, and quite sufficient (>50%) for patient #1, patient #2, patient #3. DNA methylation profiling was conducted according to the protocol approved by Bambino Gesù Children’s Hospital Ethical Committee (Protocol n° 1556_OPBG_2018, 15th January 2019). DNA was obtained from formalin fixed paraffin embedded tissues using a MagPurix FFPE DNA Extraction Kit (Resnova, Rome, Italy) for automatic extraction of genomic DNA. The samples were analyzed using Illumina Infinium Human Methylation EPIC BeadChip (EPIC) arrays (Illumina, San Diego, CA, USA), according to the manufacturer’s instructions, on Illumina iScan Platform (Illumina, San Diego, CA, USA) as previously reported [17]. Generated methylation data were compared to brain tumor classifier v11b4 [18]. High-density DNA methylation arrays allowed for determining the copy number alterations that were generated for the reported case, as described [18]. Integrative Genomic Viewer (IGV) was used for graphical visualization of structural rearrangements and mapping genes onto regions of interest.

### 2.6. RNA Sequencing

RNA was extracted from formalin-fixed paraffin-embedded neoplastic tissue. NGS analysis was performed using Archer^®^ Universal RNA Reagent Kit for Illumina^®^, Archer MBC adapters and a custom-designed Gene Specific Primer (GSP) Pool kit.

## 3. Results

### 3.1. Neuropathological and Clinical Features

All clinical-pathological features are shown in Table 1. The male/female ratio was 3:1. The median age at diagnosis was 5.5 years (range: 0, 6–9 years). Two patients were infants (2 and 0.6 years, respectively, patients #1 and #3), the other two were both nine years old children. All tumors were located in the cerebral hemispheres. Median time to recurrence was 23.5 months. In all cases, a gross total resection (GTR) was performed, both at primary and recurrence. All patients received radiation therapy (RXT) as a front-line treatment and temozolomide (TMZ) at progression, except for patient #4 who received TMZ-RXT combination therapy at primary diagnosis. According to the criteria of the 5th WHO Classification of Central Nervous System Tumors [11], paired primary and recurrent pediatric high-grade gliomas were histologically classified as follows (#1, #2, #3 and #4).

**Figure 1:**
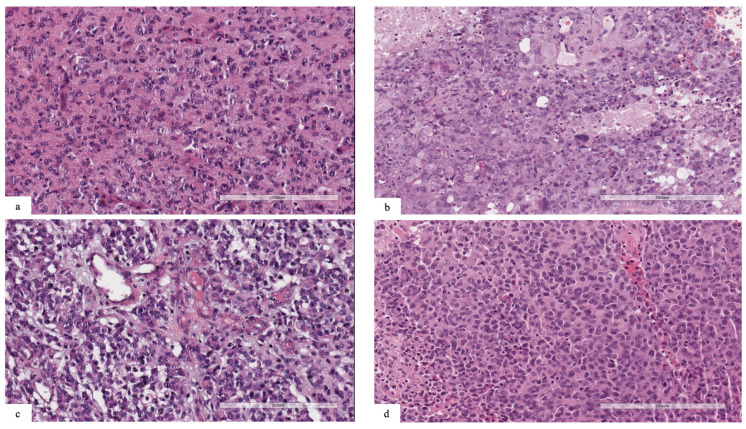
(**a**) Patient #1: neoplasm characterized by high cellularity, nuclear atypia and scant eosinophilic cytoplasm. Mitotic features were present, and necrosis and vascular proliferation were absent. (**b**) Patient #2: tumor composed of spindle, pleomorphic, epithelioid, rhabdoid and xanthomatous morphology. Eosinophilic granular bodies, mitosis (7/10HPF) and necrosis were also present. (**c**) Patient #3: neoplasm with high cellularity, nuclear atypia and scant cytoplasm. Mitotic features and vascular proliferation were present. (**d**) Patient #4: highly cellular tumor, with nuclear pleomorphism and scant eosinophilic cytoplasm. Mitotic features, necrosis and vascular proliferation were present.

Patient #1’s tumor was characterized by high cellularity, with nuclear atypia and scant eosinophilic cytoplasm. Mitotic features were present, and necrosis and vascular proliferation were absent (Figure 1a). Neoplastic cells were reactive for GFAP. The tumor was positive for the pan-TRK (EPR17341) assay (Roche/Ventana) with cytoplasmic and cellular membrane positivity. The final diagnosis was infant-type hemispheric glioma.

Patient #2’s tumor was composed of neoplastic cells with spindle, pleomorphic, epithelioid, rhabdoid and xanthomatous morphologies (Figure 1b). Eosinophilic granular bodies, perivascular lymphocytes, mitosis (7/10HPF) and focal necrosis were also present. Strong cytoplasmic immunostaining for BRAFV600E was positive in pleomorphic and spindle tumor cells. The final diagnosis was anaplastic pleomorphic xanthoastrocytoma.

Patient #3’s tumor was characterized by high cellularity, with nuclear atypia and scant cytoplasm (Figure 1c). Mitotic features and vascular proliferation were present. Neoplastic cells were reactive for GFAP and positive for pan-TRK (EPR17341) assay with cytoplasmic positivity. The final diagnosis was infant-type hemispheric glioma.

Patients #1 and #3 were both negative for the ALK (D5F3) assay.

Patient #4’s tumor was characterized by high cellularity, with nuclear pleomorphism and scant eosinophilic cytoplasm. Mitotic features, necrosis and vascular proliferation were present (Figure 1d). Neoplastic cells were reactive for GFAP and negative for IDH1 (R132H) and H3G34R/V. Tumor cells showed retained nuclear expression of ATRX protein. The final diagnosis was diffuse pediatric-type high-grade glioma, H3-wildtype and IDH-wildtype.

### 3.2. Immunohistochemistry

**Figure 2:**
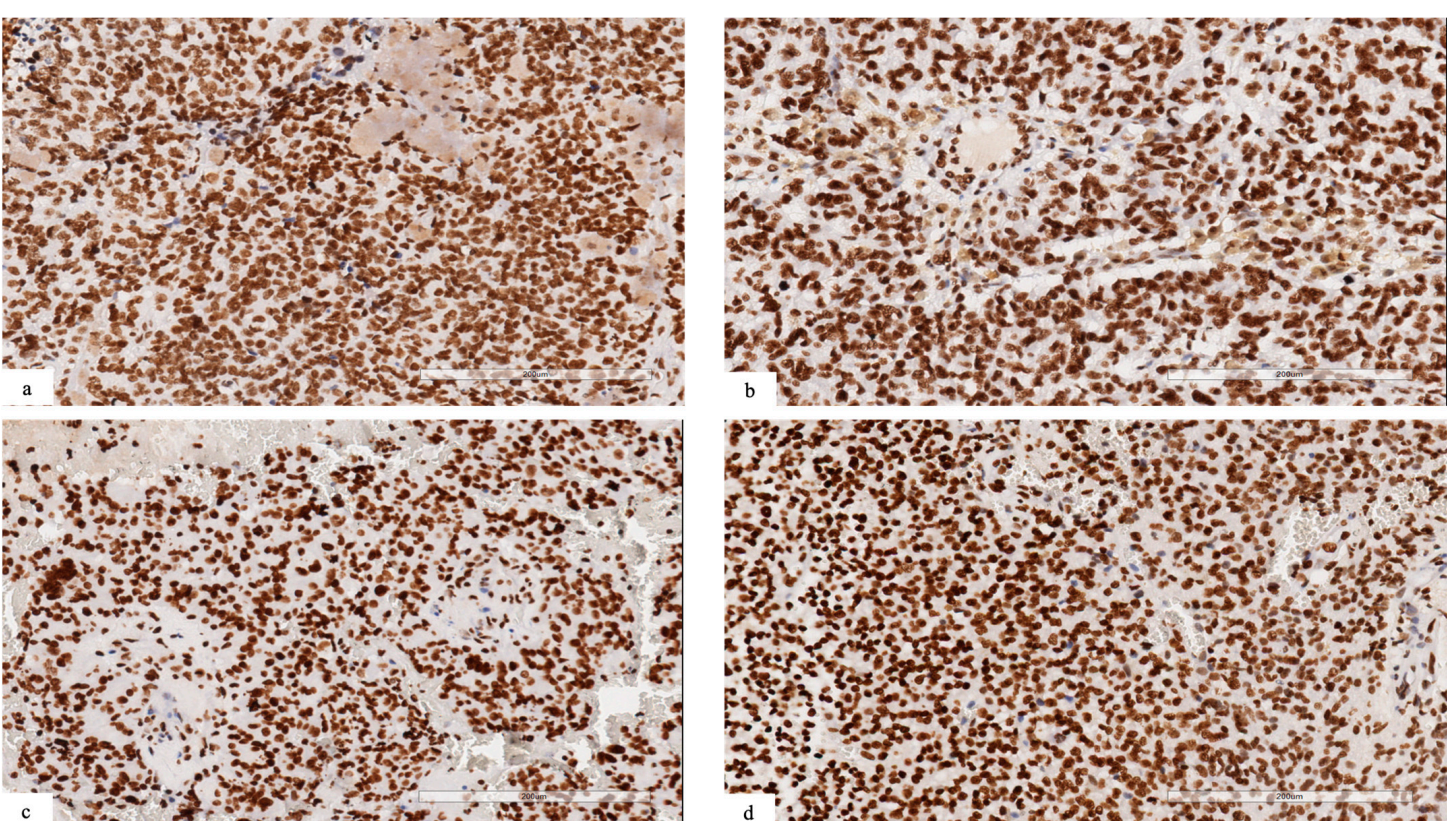
(**a**–**d**) Immunohistochemical analysis revealing retained expression for dimethyl histone H3K36 in neoplastic cells ((**a**) patient #1; (**b**) patient #2; (**c**) patient #3; (**d**) patient #4).

In cases with *NSD1* mutations, we observed a retained expression for dimethyl histone H3K36, suggesting that the methylation of H3.K36 could be regulated also by other proteins belonging to the NSD family, such as NSD2 or 3 (Figure 2a–d).

### 3.3. Identification of Somatic Mutation by Whole Exome Sequencing Analysis

WES was performed in order to characterize the genetic signature of these four tumors pairs. Samples were acquired from primary as well as recurrence and were sequenced obtaining at least a means coverage 50x. As a first approach, analysis has been focused primarily on a group of 58 specific genes that are known to be associated with pediatric HGGs, and the filter of MAF or Minority Allele Frequency (MAF < 0.05) was applied in order to exclude the polymorphisms.

**Figure 3:**
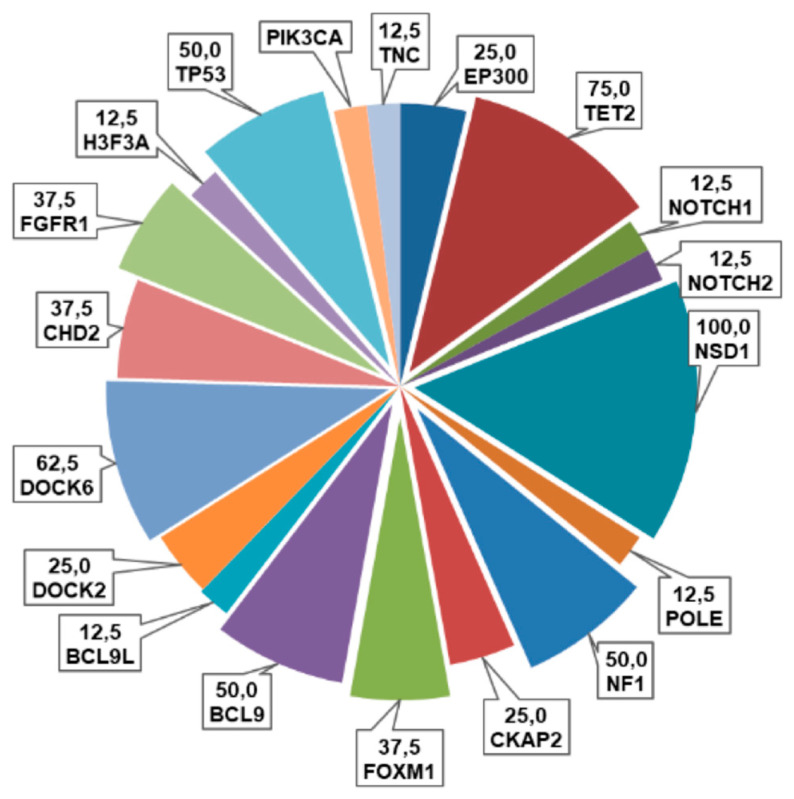
Pie chart representing the frequencies (%) of alterations of the examined genes in this cohort of pHGGs.

As shown in Figure 3, the most frequently altered genes in our cohort were *NSD1* (100%, some sample with a double variants), *DOCK6* (62.5%), *TP53* (50%), *FOXM1* and *CHD2* (37.5%), followed by *EP300* and *TET2* (25%), *H3F3A*, *FGFR1*, *PIK3CA*, *NOTCH2*, *NF1* and *CKAP2* (12.5%). Among these variants, thirty were missense (91%), two synonymous (6%) and one was a truncating variant (3%) (Figure 3).

**Figure 4:**
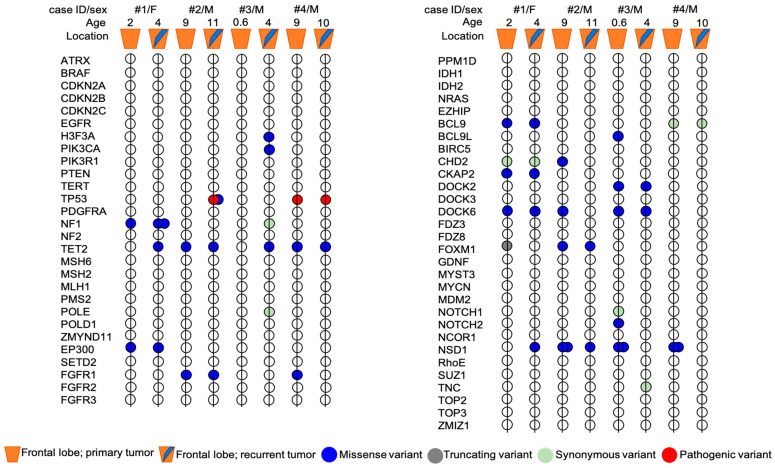
Mutational profile of primary and recurrent tumors pairs from four patients with pHGG. The exact HGVS nomenclature of each variant present in the primary tumor and/or in the relapse of each patient is shown in Figure 5. Age, sex and tumor location are indicated for each patient. Mutations are represented by colors: blue = missense, grey = truncating (frameshift), light green = synonymous, red = pathogenic.

Genetic analysis showed the presence of some variants that were shared in both primary and recurrent tumors, along with other variants exclusive of primary or recurrent tumor (Figure 4).

**Figure 5:**
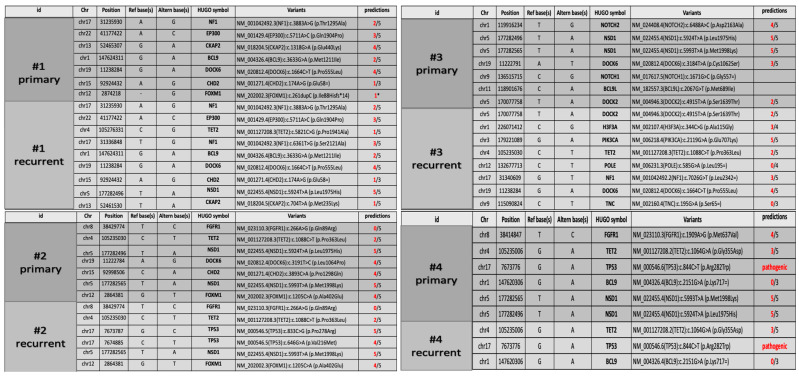
WES data collection. Candidate variants table from Whole-exome sequencing identified in four pairs of tumor samples of pHHGs. Only the variants with at least three out of five predictions of protein damage are shown in the table (except for the CDH2 variant c.174A > G, p.Glu58=, with a suspected deleterious effect on splicing).

Both primary and recurrent tumors of patient #1 shared a VUS variant of *EP300* gene (c.5711A > C; p.Gln1904Pro) and a novel *DOCK6* variant (c.1664C > T; p.Pro555Leu) already reported in COSMIC database (COSM3756305), which were predicted to be deleterious for the protein function by three out of five and four out of five in silico tools, respectively (Figure 5). Two novel variants in *CKAP2* and *FOXM1* genes were exclusive to the primary tumor. In particular, the missense variant *CKAP2* (c.1318G > A; p.Glu440Lyssilico tools. *FOXM1* (c.261dupC) was a truncating variant localized in the N-terminal autorepressor domain (negative regulatory domain, NRD) that causes the premature termination at codon 101. The novel *NF1* variant (c.6361T > G (p.Ser2121Ala) and *NSD1* variant (c.5924T > A; p.Leu1975His), occurring within the highly conserved SET domain, were instead exclusively identified in the recurrent tumor. The prediction analysis for the proteins function foresaw that both these mutations have a high probability to cause a protein damaging by three out of five and five out of five in silico tools, respectively (Figure 5).

In patient #2’s tumors pair, genetic analysis identified three novel variants exclusive of the primary tumor: the variants are located in the genes *DOCK6* (c.3191T > C; p.Leu1064Pro), *CHD2* (c.3893C > A; p.Pro1298Gln) and *NSD1* (c.5924T > A; p.Leu1975His). Interestingly, the *NSD1* variant (c.5924T > A; p.Leu1975His) was also identified in the recurrent tumor of patient #1. The prediction analysis for *DOCK6* and *CHD2* variants, although the variants were not localized in the functional domains of the proteins (https://www.uniprot.org/uniprot/Q96HP0#family_and_domains (accessed on 27 September 2021)); https://www.uniprot.org/uniprot/O14647#family_and_domains (accessed on 27 September 2021)), estimated a strong probability to be deleterious for the protein function (four out of five in silico tools) for both variants (Figure 5). Primary and recurrent tumor also shared a variant in *FOXM1* gene (c.1205C > A; p.Ala402Glu) and a novel variant in *NSD1* gene (c.5993T > A; p.Met1998Lys), localized within the highly conserved SET domain, and was predicted to be deleterious for the protein function by four out of five and five out of five in silico tools, respectively. The presence of two missense variants of *TP53* in the recurrent tumor assumes particular significance in this case. Patient #2’s age was nine years at first diagnosis and eleven years at recurrence. The mutational rate of *TP53* gene is high in HGGs and the frequency of *TP53* variants is directly proportional to the age of the patients (The Cancer Genome Atlas database (https://portal.gdc.cancer.gov/ (accessed on 28 September 2021)). Both the *TP53* variants (c.833C > G; p.Pro278Arg; c.646G > A, p.Val216Met) were missense with still unknown and debated clinical significance (Figure 5). The Varsome and IARC TP53 databases reported both these variants as pathogenic and responsible for protein alteration, while the ClinVar database considered them as Uncertain, although the majority of submitters classify these variants as Likely Pathogenic or Pathogenic. The variants cause alteration in the DNA-binding domain of the protein, which is the site of several oncogenic mutations responsible of p53 loss of function. In silico prediction tools for both foresaw that these variants are deleterious for the protein function (five out of five and four out of five in silico tools, respectively).

The analysis of patient #3’s tumors pair did not identify shared variants between the primary and the recurrent tumor. *NSD1* variants (c.5924T > A; p.Leu1975His and c.5993T > A; p.Met1998Lys), together with the *NOTCH2* variant (c.6488A > C; p.Asp2163Ala), the *DOCK6* variant (c.3184T > A; p.Cys1062Ser) and the *BCL9L* variant (c.2067G > T; p.Met689Ile), have been identified only in the primary tumor. Bioinformatics tools predicted that these variants, novel and not yet reported in any databases, can be deleterious to protein function (Figure 5). Recurrent tumor presented the following set of mutations: *H3F3A* (c.344C > G; p.Ala115Gly), *PIK3CA* (c.2119G > A; p.Glu707Lys), *NF1* (c.7026G > T; p.Leu2342=) and *DOCK6* (c.1664C > T; p.Pro555Leu) (Figure 5). For all of them, in silico prediction tools estimated a high probability of them to be deleterious for the protein function (Figure 5). Interestingly, the *NF1* synonymous variant could be related to altered splicing process, as suggested by the bioinformatic tools.

In the patient #4’s tumors pair, a pathogenic variant of *TP53* (c.844C > T; p.Arg282Trp) was identified both in the primary and in recurrent tumor with an allele frequency of 85 and 91%, respectively. The variant was associated with Li–Fraumeni syndrome, which was subsequently diagnosed in the family. Primary and recurrent tumors shared also a *TET2* variant (c.1064G > A; p.Gly355Asp) (3 out of 5 in silico tools). Moreover, only in the primary tumor, genetic analysis identified the already described *NSD1* variants (c.5924T > A; p.Leu1975His and c.5993T > A; p.Met1998Lys) and a *FGFR1* variant (c.1909A > G; p.Met637Val), with a high prediction to be deleterious for the protein function and for the protein kinase domain role of FGFR1 (Figure 5). To summarize, *NSD1* mutations were present only in the primary tumor in two out of four patients (#3 and #4), conserved over time in one out of four patients (#2) and acquired at the recurrence in one out of four patients (#1).

### 3.4. DNA Methylation Analysis and Copy Number Variation (CNV) Analysis

The genome-wide methylation data of the tumors were categorized using the brain tumor classifier v11b4 (https://www.molecularneuropathology.org/mnp/classifier/2 (accessed on 25 March 2021) [18], which also generated a copy number variation (CNV) plot.

Despite the accurate selection of tumoral areas, residual neoplastic cell content was <70%, except for patient #4, whose tumor content was >70%.

For patients #1, #2 and #3, both primary and recurrent tumors clustered in the methylation class of family plexus tumors, subclass pediatric (PLEX, PED B), with low calibrated scores (<0.5), while patient #4’s specimens did not cluster with any recognized methylation class. Looking at raw scores, the first and second methylation class were, respectively, control tissue hemispheric (CONTR, HEMI) and hemangioblastoma (HMB) for both primary and recurrent tumors of patients #1 and #3 and for primary tumor of patient #2. Conversely, for patient #2’s recurrent tumor, the first methylation class was control tissue inflammatory tumor microenvironment (CONTR, INFLAM) and the second one was control tissue hemispheric (CONTR, HEMI). Patient #4’s tumors had very low raw scores, with the first methylation class of glioblastoma IDH wildtype, subclass RTK III (GBM, RTK III) and second one of melanoma (MELAN) (Supplementary Figure S1).

Altogether, these data suggest that DNA methylation profiling could not confirm nor orient the diagnosis for case #1, #2 and #3, likely due to the low content of neoplastic cells. On the other hand, the low calibrated score could also be related to the inexistence of a well-defined entity in the current version of the classifier.

Indeed, it has been reported that tumors that cannot be correctly classified (with a score > 0.9) are often classified as PLEX PED B, even though there is no clear connection to this tumor group. Classifier developers suggest that a low score for PLEX PED B, in an unexpected setting, should therefore be interpreted with extreme caution [18].

Once available, the v12.3 brain tumor classifier was also used to further categorize the methylation data of the tumors (data not shown). Patient #1’s primary tumor showed low raw scores (0.56) in the methylation class of glioblastoma, pediatric RTK1 type, subtype B, while the recurrent tumor showed very low raw scores (0.15) in the methylation class of anaplastic pleomorphic xanthoastrocytoma. Patient #2 showed, for the primary tumor, high raw scores (0.88) in the methylation class of pleomorphic xanthoastrocytoma, and, for the recurrent tumor, in the methylation class of glioblastoma, mesenchymal subtype, subclass B for the recurrent (0.78).

Primary and recurrent tumors from the patient #3 showed low raw scores (0.005 and 0.51) in the methylation class of glioblastoma, pediatric RTK1, subtype B and of diffuse leptomeningeal glioneuronal tumor subtype 1, respectively.

Finally, both of patient #4’s primary and recurrent tumors showed very low raw scores in the methylation class of glioblastoma, pediatric RTK2 type, subtype B (0.12 and 0.33).

With regards to CNV evaluation, deletion of CDKN2A/B was a common feature of all analyzed primary and recurrent pHGGs. Patient #1 also showed chromosome 1p loss and chromosome 10 loss in both samples. For patient #4, gains of chromosome 1, 2, 7, 10, 11, 15, 16, 20, 21 and X were detectable in primary and recurrent tumors, together with a small deletion spanning chr3:50,293,775–50,703,190 and including, among the others, SEMA3B, HYAL3, CACNA2D2 and MAPKAPK3. We also looked specifically at mapping regions of the genes with missense variants (*TP53*, *NF1*, *TET2*, *NSD1*, *DOCK6*, *BCL9*, *FGFR1*, *FOXM1*, *CHD2*, *H3F3A*, *PIK3CA*, *POLE*, *BCL9*, *BCL9L*, *NOTCH1* and *TNC*), but no gain or loss were identified (Supplementary Figure S2).

### 3.5. RNA Sequencing

A novel in frame *KCTD8::NTRK2* fusion, involving exon 1 of *KCTD8* and exon 16 of *NTRK2*, was identified in patient #1. The breakpoints were at chr4:44,449,580 and chr9:87,482,158 for *KCTD8* and *NTRK2*, respectively. The gene fusion was confirmed by RT-PCR and Sanger sequencing.

## 4. Discussion

Pediatric high-grade gliomas have a median overall survival of 9–15 months [19,20,21], representing the most common malignant brain tumors and the greatest cause of cancer-related death under the age of 19 years [19,20,21]. Unlike similar lesions in adults, which tend to be restricted to the cerebral hemispheres, pHGGs can occur throughout the CNS [22].

This heterogeneous group of tumors comprises circumscribed and diffuse high-grade gliomas with a wide variety of molecular features. However, little is known about their temporal and therapy-related genomic heterogeneity. An adequate understanding of the evolution of pHGGs genomic profiles over time is critically important in guiding decisions about targeted therapeutics and diagnostic biopsy at recurrence.

Emerging data suggest variable temporal genomic heterogeneity across other pediatric central nervous system (CNS) tumors. In medulloblastoma, molecular subgroup is conserved [23], but there is significant divergence in targetable mutations between primary and recurrent. Transcriptomic changes between matched primary and recurrent pediatric posterior fossa ependymomas have been reported with relative preservation of copy number alterations [24]. In pHGGs, a study of temporal genomic heterogeneity across 16 paired pediatric HGGs demonstrated conservation of certain key driver mutations at recurrence, but acquisition or loss of others [16].

To determine the temporal stability of the mutational profile of pHGGs, we performed WES on four tumor pairs (primary and recurrence), all located in the frontal lobe. Our aim was to determine if mutations were stable between primary and recurrence, or if recurrent tumors could present sub-clonal mutations related to treatment or progression.

In our series, we observed the presence of some variants being shared between primary and recurrent tumors, and other variants that were exclusive of primary or recurrence. Furthermore, we identified a mutation frequency greater than 50% in *NSD1*, *DOCK6* and *TP53*.

The NSD family (nuclear receptor-binding SET domain protein) is a group of histone methyltransferases (HMTases) comprised of: NSD1, NSD2 (MMSET/WHSC1), and NSD3 (WHSC1L1). NSD1 is a histone lysine methyltransferase, with functions of mono- and di-methyltransferase, targeting histone H3 at K36 (H3.K36). Among the mechanisms of epigenetic regulation, histone methylation and its regulation by histone methyltransferases and demethylases has emerged as a particularly interesting subject in recent studies [25,26].

The NSD proteins are important in multiple aspects of development and disease. Pathogenic *NSD1* mutations have a genome-wide impact on DNA methylation, generating a specific episignature, and are involved in Sotos syndrome, Wolf–Hirschhorn syndrome and different types of cancer [27]. *NSD1* mutations have been implicated in the pathogenesis of acute myeloid leukemia [28], HPV-negative head and neck squamous cell carcinoma [29], hepatocellular carcinoma [30] and colorectal carcinoma [31]. Most *NSD1* missense mutations are present in the functional domains of NSD1 protein, and to date, a total of 21 missense mutations have been identified in the SET domain [32]. Although the SET domain has a crucial role on the histone methyltransferase activity of NSD1, the mechanisms by which these mutations result in loss-of-function have been poorly understood.

*NSD1* mutations detected in our study occur within the highly conserved SET domain and have a high probability to cause protein damage, as predicted by in silico tools. The novel *NSD1* variants (c.5924T > A; p.Leu1975His and c.5993T > A; p.Met1998Lys) were present at high frequency in our series (100%; sometimes associated in double mutants). The novel *NSD1* variant c.5924T > A (p.Leu1975His), occurring within the highly conserved SET domain, was exclusively identified in the recurrent tumor in patient #1 and in the primary tumor in patients #2, #3 and #4. The prediction analysis for the protein function foresaw that this mutation has a high probability to cause a protein damage. In patient #2, the novel *NSD1* variant c.5993T > A (p.Met1998Lys) was present in both primary and recurrent tumors. Patients #3 and #4 were carriers of the novel *NSD1* variant c.5993T > A (p.Met1998Lys), but exclusively in the primary tumor.

As NSD1 is a mono- and di-methyltransferase targeting H3.K36, we examined DiMethyl-H3.K36 expression by immunohistochemistry. Despite the presence of *NSD1* mutations, we observed a retained expression of DiMethyl-H3K36, suggesting that H3.K36 methylation could also be regulated by other NSD proteins, such as NSD2 or 3.

Chromatin modifying enzymes are frequently mutated in cancer, resulting in widespread epigenetic deregulation. Since NSD1 is a methyltransferase, these tumors may have a completely different epigenetic profile, explaining the fact that tumors from patients #1, #3 and #4 do not classify with any known entity at DNA methylation analysis. However, patient #1 showed immunopositivity for pan-TRK (Roche/Ventana) and a novel in frame *KCTD8::NTRK2* fusion, involving exon 1 of *KCTD8* and exon 16 of *NTRK2* by RNA sequencing, in part coherent with the methylation findings of the v12.3 brain tumor classifier (with the bias of the low tumor content).

In our study, we also identified novel mutations involving *DOCK6* gene. Dedicator of cytokinesis 6 gene (*DOCK6*) encodes an atypical guanidine exchange factor (GEF), which activates two members of the Rho GTPase family, Cdc42 and Rac1. Mutations in gene *DOCK6* are associated with Adams–Oliver syndrome type 2 [33]. *DOCK6* has been found to be mutated in acute myeloid leukemia [34] and has been reported to be highly expressed in gastric cancer [35], correlating with tumor size, depth of invasion, lymph node metastasis, vascular invasion and pathological stage. This is the first study reporting *DOCK6* mutations in gliomas. The few studies investigating the biological functions of *DOCK6* pointed at its role in the nervous system: its expression is induced upon neuronal differentiation, being fundamental for axonal growth [36]. In our series of pHGGs, *DOCK6* mutations were identified exclusively at the primary tumor in two patients (#2 and #3), conserved over time in only one patient (#1) and acquired only at relapse in patient #4, suggesting a possible involvement in progression, as already documented in literature for gastric cancer. Moreover, in our study, we identified the *DOCK6* variant c.1664C > T (p.Pro555Leu), already reported in COSMIC database (COSM3756305) and predicted to be deleterious for the protein function by four out of five in silico tools. In patient #1, this variant was present both in primary and relapse tumor, while in patient #3 in exclusively the recurrence. Other novel *DOCK6* variants (c.3191T > C; p.Leu1064Pro and c.3184T > A; p.Cys1062Ser) were identified in the primary tumors of patient #2 and #3, respectively.

Finally, in our study some variants of *TP53* have also been identified. All of them were located in the DNA binding domain, which is the site of several oncogenic mutations. Hence, they may be potentially involved in the loss of p53 ability to interact with specific DNA promoters (loss of function). Specifically, in patient #2, we identified, exclusively in the recurrent tumor, the *TP53* variants c.833C > G (p.Pro278Arg) c.646G > A (p.Val216Met). Instead, in patient #4, belonging to a family with post-diagnosed Li–Fraumeni syndrome, the same *TP53* pathogenic variant c.844C > T (p.Arg282Trp) has been found in both primary and relapse tumor.

In order to exclude structural variations, we also looked specifically at mapping region of the genes with missense variants (*TP53*, *NF1*, *TET2*, *NSD1*, *DOCK6*, *BCL9*, *FGFR1*, *FOXM1*, *CHD2*, *H3F3A*, *PIK3CA*, *POLE*, *BCL9*, *BCL9L*, *NOTCH1* and *TNC*), but no gain or loss were identified.

## 5. Conclusions

Our study highlighted the presence of specific sub-clonal mutations in the progression of pHGG. The presence of *NSD1* mutations that only occur at recurrence suggest that they can be sub-clonal, while the presence in both primary and recurrence implies that they can also represent early and stable events. Furthermore, their presence in primary, but not in the recurrent tumor, suggests that *NSD1* mutations may also be influenced by treatment. Multiple factors could contribute to this variability, including pharmacodynamics and heterogeneity within the tumor and between tumor and microenvironment [37]. The relationship between clonal heterogeneity and clinical significance of sub-clonal driver mutations is only recently beginning to be explored across brain tumors. Additionally, our study identified novel *NSD1* mutations which may play a role in pHGGs pathogenesis, progression and relapse. Nevertheless, these findings deserve further investigations, and, as a future perspective, is our intention to better understand the role of *NSD1* in the different types of pHGGs by also examining its implications in the therapeutic setting.

## Figures and Tables

**Table 1:** Neuropathological and clinical features. Abbreviations: HGG: high grade glioma; PXA: xanthoastrocytoma; NED: no evidence of disease; GTR: gross total resection; RXT: radiotherapy; TMZ: temozolomide.

	Sex/Age	Location/Diagnosis	Status at Last Follow-Up	Resection	Therapy
Patient #1 primary	F/2	Frontal/infant-type hemispheric glioma		GTR	RXT
Patient #1 recurrent	F/4	Frontal/infant-type hemispheric glioma	Alive, NED	GTR	TMZ
Patient #2 primary	M/9	Frontal/anaplastic PXA		GTR	RXT
Patient #2 recurrent	M/11	Frontal/anaplastic PXA	Alive, NED	GTR	TMZ
Patient #3 primary	M/0,6	Frontal/infant-type hemispheric glioma		GTR	RXT
Patient #3 recurrent	M/4	Frontal/infant-type hemispheric glioma	Alive, NED	GTR	TMZ
Patient #4 primary	M/9	Frontal/pediatric-type high-grade glioma, H3- and IDH-wildtype		GTR	RXT + TMZ
Patient #4 recurrent	M/10	Frontal/pediatric-type high-grade glioma, H3- and IDH-wildtype	Died at progression		

## Data Availability

Not applicable.

## Supplementary Materials

The following supporting information can be downloaded at: https://www.mdpi.com/article/10.3390/diagnostics13010078/s1, Figure S1: DNA methylation class and raw score of each patient. Figure S2: Copy Number Variation (CNV) analysis of each patient.
