# Supplementary figures and images for: NSD1 Mutations and Pediatric High-Grade Gliomas: A Comparative Genomic Study in Primary and Recurrent Tumors
Source: Diagnostics (Basel). 2022 Dec 27;13(1):78. doi: 10.3390/diagnostics13010078 (PMC9818856; doi:10.3390/diagnostics13010078)

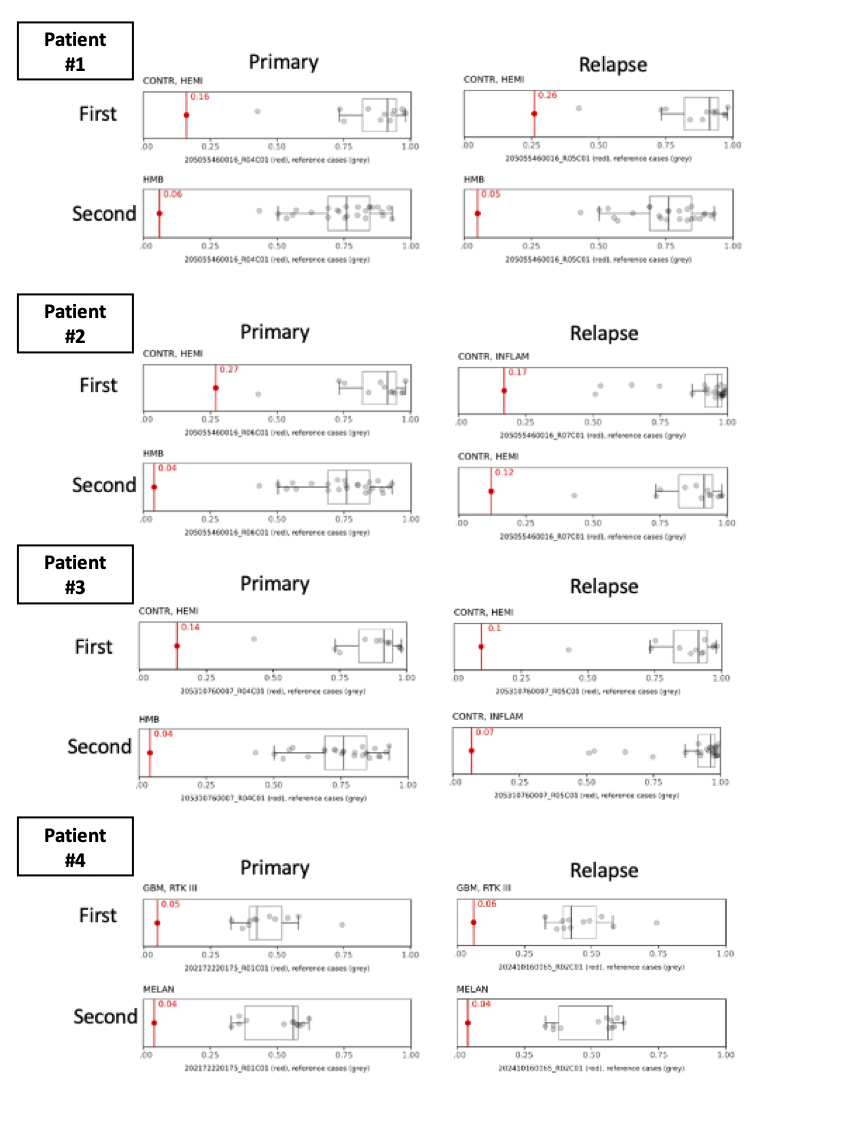

Supplement: Supplementary file 1 [file diagnostics-13-00078-s001.zip › Supplementary Figure S1.png]

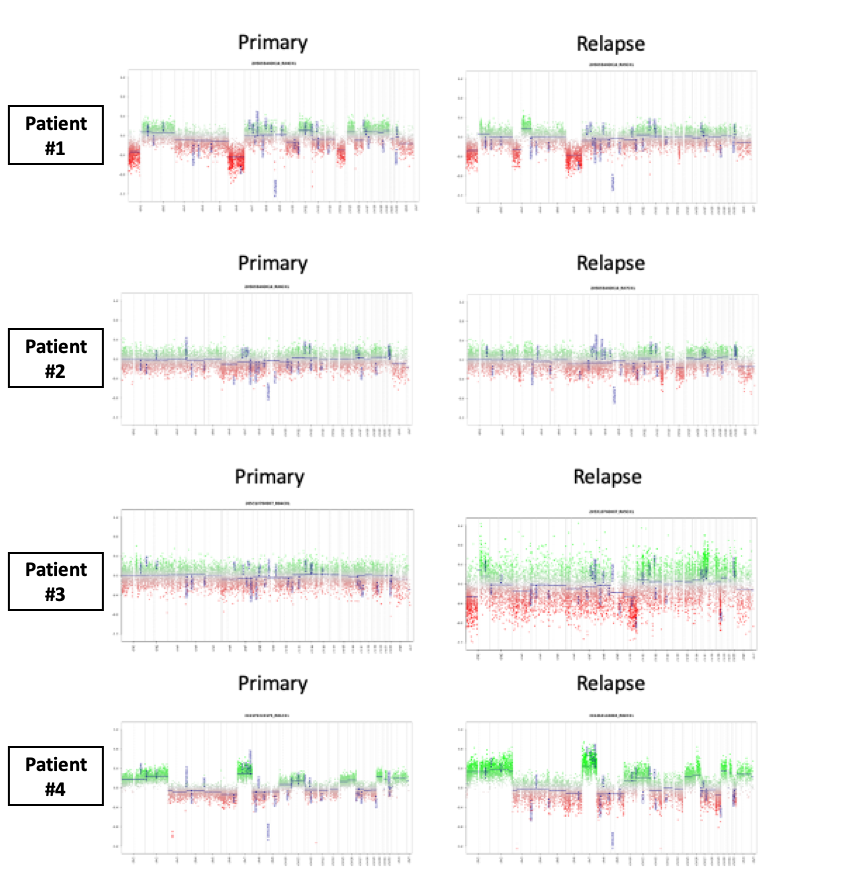

Supplement: Supplementary file 1 [file diagnostics-13-00078-s001.zip › Supplementary Figure S2.png]
